# Impacts of family care for children and the elderly on women’s employment: evidence from rural China

**DOI:** 10.3389/fpsyg.2023.1208749

**Published:** 2023-09-15

**Authors:** Xinru Miao, Jiqin Han, Shaopeng Wang, Bing Han

**Affiliations:** ^1^College of Economics and Management, Nanjing Agricultural University, Nanjing, China; ^2^Faculty of Agriculture, Universiti Putra Malaysia, Serdang, Selangor, Malaysia

**Keywords:** family care, working outside, public services, rural women, gender equality

## Abstract

**Introduction:**

China’s traditional culture makes rural women and men take on different family responsibilities.

**Methods:**

Use “China Family Panel Studies” (CFPS) data and build Logit and propensity score matching models to empirically study the impact of children care and elderly care on rural married women going out to work. And explore the welfare effects of basic education public services in helping rural women take care of their families.

**Results:**

The results show that caring for children has a significant hindering effect on rural married women’s job hunting. Especially for those in low-income families, the employment inhibition is most significant among women aged 20–30 with multiple children. Contrary to previous cognition, supporting the elderly has a certain weak stimulating effect. The kindergarten public services in rural areas can help women take care of their children and relieve their work pressure. The primary school public services have not played a role in alleviating them.

**Discussion:**

This shows that there are still a large number of female laborers in rural China who are unable to go out to work due to family care. The improvement of rural basic education public services can promote more rural women going out to work. This finding will provide a policy reference for the introduction of a formal care system and the establishment of basic education public services in China.

## 1. Introduction

Family care and rural female labor participation has always been an important social issue. In the family, women play the responsibility of motherhood role and also take the responsibility of caring for other family members (Lachance-Grzela and Bouchard, 2010). With economic and social development, more and more women are taking on the responsibility of both family and work (Bianchi et al., 2012). Constrained by limited time and energy, this poses new challenges for women in finding a balance between work and family care (Budig and England, 2001). Women’s labor force participation provides the economic foundation for gender equality, but it also exposes them to conflicts between family and work (Craig, 2006). Globally, there is a large proportion of women who are excluded from the labor force due to family care, while the proportion of men is low (Gornick and Meyers, 2008; Kabeer, 2010). Among them, young mothers from rural areas, especially married women with a high number of children, spend more time on family care (Zhang, 2011; Zhu, 2011; Gu, 2020). The apparent squeeze on rural women’s personal development as a result of caring for their families means that female workers take on a large amount of unpaid labor, which may negatively affect women’s physical and mental health (Craig and Mullan, 2010). Unpaid caregiving responsibilities not only take up a significant amount of rural women’s time, but also reduce their employment opportunities and income (Gornick and Meyers, 2008). Thus, family care arrangements have a direct impact on gender equality, rural women’s development, and household economic levels. In order to support their personal career development and alleviate the time conflict between work and family roles, it is urgent for government agencies and social organizations to adopt policies that support rural women in achieving work-family balance.

Since the implementation of the family planning policy in the late 1970s in China, the population has been controlled and the population structure has also undergone tremendous changes. The proportion of elderly people aged 65 and over has increased from 4.67% in 1980 to 14.20% in 2021, and the newborn birth rate has dropped from 18.21‰ in 1980 to 7.52‰ in 2021 (National Bureau of Statistics of China, 2022). The United Nations predicts that in 2030, the proportion of China’s elderly population over 60 in the total population will rise to 25.3%, and it will be as high as 36.5% in 2050. This proportion is higher than that of the United States and comparable to most developed countries in Europe. In response to an aging population and labor shortages in the future, China has enacted a “three-child policy” to encourage young couples to have more children. However, due to the heavy burden of raising newborn children and supporting the elderly, and the miniaturization of family scales has been generally accepted by Chinese society, it will be difficult to fundamentally reverse the shortage of labor in the future.

**FIGURE 1:**
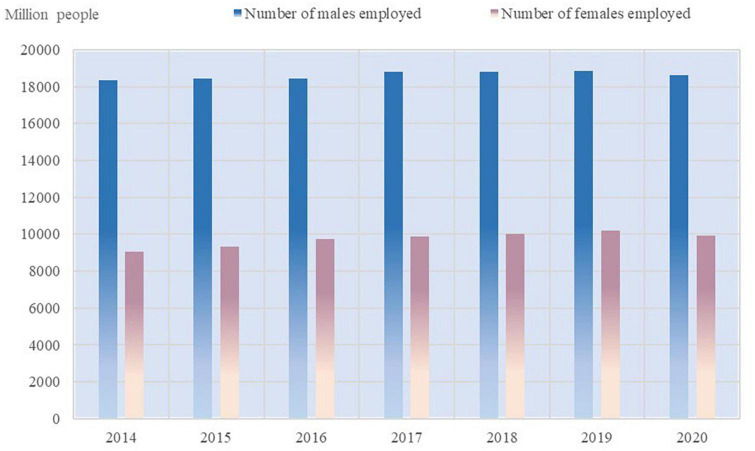
2014–2020 rural labor employment. Source: Department of Rural Socio-Economic Survey, National Bureau of Statistics of China (2014-2020).

According to the latest data from the Seventh National Census (SNC), the rural female population is only about 6% smaller than that of males (National Bureau of Statistics of China, 2022). However, Figure 1 shows that the number of rural women going out to work in China is only about 50% of that of men, and the absolute number of employed women is much smaller than that of men. According to World Bank statistics, China’s female labor force participation rate has been declining year by year since 1990 when it was 73.24%. By 2021, the female labor force participation rate in China is 44.51%, compared to 46.27% in the US and 46.34% in the EU, which is still a gap with developed countries such as Europe and the US. In terms of gender wage gap, the gender wage gap in most regions of the world shows a convergence trend, but the gender wage gap in China’s labor market shows a widening trend. According to the World Inequality Report 2022, Figure 2 shows that the share of female labor income in total labor income in China has decreased from 39.14% in 1991 to 33.41% in 2019. However, the share of female labor income in total labor income in most parts of the world is on the rise. Based on the types of female jobs, Figure 3 shows that the gender wage gap for Chinese women in part-time jobs is 23.6, which is higher than full-time jobs. Due to the low level of education and lack of highly technical professional skills on average (Gu et al., 2021), Chinese rural women are more likely to work part-time with low professional skills than in full-time jobs with high skill levels. The inclusion of rural women in the labor market is a result of the pursuit of gender equality and the practice of “work-family” among China (Stier et al., 2012). Only by creating more wealth can women affirm their value and achieve economic independence.

**FIGURE 2:**
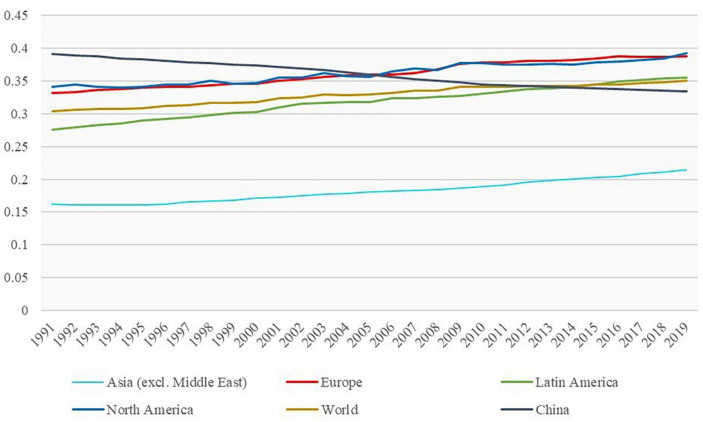
1991–2019 Female labor income share. Source: World Inequality Database.

**FIGURE 3:**
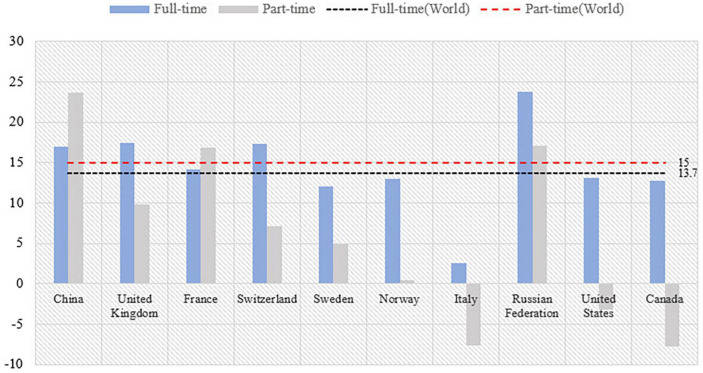
2018/19 Gender wage gap between full-time and part-time employment. Source: Global Wage Report (2018/19).

What are the reasons that restrict rural women’s employment? Different from the family cultures in western countries, East Asian family cultures are characterized by strong social ties between family members of multi-generational lineages (Oh and Lee, 2014). Especially in traditional Chinese rural families, three generations often live together. The family relationship generated by this form of living makes women inevitably take care of the elderly and children (Voydanoff, 2004; Ji et al., 2017). The care responsibility, which is closely related to women, is an important factor restricting women’s employment (Dex and Bond, 2005; Kinnunen et al., 2006; Cook and Dong, 2011; Qi and Dong, 2018). If the basic public services related to family care in rural areas are improved, the pressure of family care for rural women are alleviated, and it may promote women’s participation in work (Huffman and Lange, 1989; Lippe and Dijk, 2002).

According to the economic and social theory, rural women’s biological characteristics give them a comparative advantage in household production. As a result, rural men typically take on the economic responsibility of providing for their families, while women take on the domestic responsibilities of caring for children and the elderly (England, 2010; Budig et al., 2012). Although modern society has provided many forms of caregiving resources in rural areas, childcare responsibilities are still firmly tied to women (Leigh, 2010). Traditional gender culture emphasizes the importance of mothers caring for their children, believing that only mothers can provide healthy and comprehensive care for their children (Lee and Brann, 2015). When a mother’s employment conflicts with her children’s upbringing, women are constrained by cultural traditions and tend to lose their jobs, take pay cuts or demotions in exchange for more time to provide the best care for their children (Misra et al., 2007; Budig and Hodges, 2010). In China, rural women bear most of the responsibility for caring for the elderly and children due to traditional beliefs. So, how does family care affect rural women’s employment? Based on the cost-benefit principle, rural women are expected to reallocate the time required for family care and employment as needed, which can be seen as allocating time between paid market work and unpaid family care work (MacPhail and Dong, 2007). A growing body of research on the relationship between caregiving and rural female employment supports the concept of time conflict. Studies have found that providing family care leads rural women to work fewer hours (Spieß and Schneider, 2003; Houtven et al., 2013; Mengy, 2013). That is, rural women who need to spend more time on family care are at high risk of not being able to strike an appropriate balance between family and work. They will adjust their employment status (Heitmueller, 2007; Berecki-Gisolf et al., 2008; King and Pickard, 2013; Kelle, 2020).

In family care, different family members have different time constraints on women, leading to the fact that caring for different family members can have different effects on their employment. On the one hand, there is a strong emotional bond between rural women and the elderly. As China’s population ages, rural women are influenced by the traditional division of gender roles in the family, resulting in them taking on most of the caregiving responsibilities for the elderly (Casado et al., 2011; Chen and Fan, 2016). Some studies have shown that caring for the elderly significantly reduced rural women’s labor force participation rate for their employment (Bolin et al., 2008; Liu et al., 2010; Huang, 2012; Wu et al., 2017). However, other studies showed that the impact of caring for the elderly on rural female employment depended on the physical condition of the elderly (Liu and Zhang, 2018). Even some elders can provide intergenerational care (Attias-Donfut et al., 2005; Hank and Buber, 2009). This gives rise to the interesting fact that the elderly play dual roles as both care recipients and helpers for women in childcare. It is clear that caring for the elderly sometimes does not reduce rural women’s work possibilities and hours, as studies have confirmed using data from Spain and other European countries that caring for the elderly did not reduce rural women’s work hours (Bolin et al., 2008; Casado et al., 2011). On the other hand, based on the theory of division of family roles, mothers are considered ideal childcare recipients. Raising children is an activity that requires a great deal of time (Yu and Xie, 2014). In the fierce social competition, mothers are busy working and inadequate childcare has become a common problem in modern societies (Doiron and Kalb, 2005; Viitanen, 2005; Mahringer and Zulehner, 2015).

To alleviate female caregiving constraints, existing studies have mainly examined the role of informal caregiving as a substitute for rural female childcare. Intergenerational caregiving is a common form of informal caregiving used by rural families. As a form of intergenerational reciprocity, it is an important way to increase rural women’s labor force participation. Intergenerational care can, to some extent, help rural women balance work and family. This type of caregiving is less costly (Du et al., 2018). Both rural men and women participate in the labor market in order to increase family income. The demand for intergenerational care is further increased especially when the elderly are in good health (Zou et al., 2018). As public services for basic education become more widely available in rural areas, rural children can also choose kindergartens and elementary school to alleviate female care constraints. Childcare in public education institutions is specialized and regulated. It becomes an important alternative to female caregiving (Fitzpatrick, 2010). However, the existing literatures are inadequate in analyzing the role of public services in alleviating rural female time constraints. Therefore, there is a need to analyze whether formal care institutions based on public education institutions can alleviate the time constraints as well as increase the work possibilities of rural females. This would fill the gaps in existing research.

The existing literatures analyzed the impact of caring for the elderly and children on rural women’s work from the perspectives of the theory of division of family roles, which provides the basis for further analysis in this paper. However, the existing literatures suffer from several deficiencies: First, traditional gender division of labor is prominent in rural areas, but limited literatures examined the impact of family care on the employment of married women in rural areas. Furthermore, there is scarce literature that integrates the caregiving of the elderly and children into a unified framework, making it difficult to directly compare the impacts of caregiving for the elderly and children on rural women’s employment. Finally, most studies are focused on analyzing the mechanisms of informal caregiving, such as intergenerational caregiving and father involvement in childcare, in alleviating family stress for rural women. However, there have been few studies based on social support theory that have examined the role of basic education public services in alleviating the challenges faced by women when balancing family and work responsibilities. Therefore, based on the theory of division of family roles, social support theory and time allocation theory, this paper examined the effects of informal caregiving on the employment of married women in rural areas, considering the dual perspective of family care. Furthermore, it explored how the provision of basic education public services can enhance work opportunities for rural women. By doing so, it aims to unleash the labor potential of rural women and address the labor shortage challenges resulting from China’s ongoing population aging.

We did the following explorations. First, by employing econometric methods such as Logit regression and propensity score matching, this paper examined the differential effects of caregiving for the elderly and caregiving for children on the employment of rural women. Second, recognizing the bidirectional causality between childcare and women’s employment, instrumental variable methods were employed to address endogeneity issues in the model. Third, robustness analyses were conducted by replacing explanatory variables and employing different model specifications to assess the stability of the relationship between childcare and women’s employment. Fourth, the magnitude of the impact of childcare on women’s employment varied depending on women’s age, number of children, and household income. Heterogeneity analysis was performed through regression analysis on subgroups. Finally, this paper explored the moderating role of basic education public services in the relationship between childcare and women’s employment in rural areas. By comprehensively examining the interplay among family care, basic education public services, and women’s employment, it was demonstrated that improving the level of rural basic public services can help mitigate the impact of caregiving responsibilities. This, in turn, encourages rural women to seek employment and facilitates the release of more labor force.

## 2. Conceptual framework

Family care is a family behavior rooted in human instinct. However, in modern society, family care is influenced by social, economic, and cultural factors, giving it a distinct social dimension (Hook, 2010). When rural women face economic pressures and need to work, they often have to make choices in allocating their time between family care and employment (Craig and Mullan, 2010; Bianchi et al., 2012). As daily household expenses increase and the number of children grows, the conflict between the time constraints of family and work becomes more pronounced (Lee and Brann, 2015). It is crucial to conduct a thorough analysis of the logical relationship between family care and rural women’s employment, following the fundamental principles of sociological and relevant economic theories.

According to the theory of division of family roles, there are gender differences in the domains of work and family, and a rational division of labor based on gender can lead to higher economic efficiency (Bianchi and Milkie, 2010). However, influenced by historical and cultural traditions, rural women are often assigned the responsibility of family care, while men are expected to provide income (England, 2010). Nevertheless, given the changing dynamics of modern family structures and the development of the theory of division of family roles, it is recognized that women’s participation in the workforce is their fundamental right. However, even as rural women enter the labor market, they still bear the primary responsibility for family care (Gornick and Meyers, 2008; Sayer and Gornick, 2012). The interplay between family care and work choices involves a series of balancing decisions for rural women under the pressure of their dual roles.

As education levels rise, an increasing number of families choose to place their children in daycare centers or schools. Based on time allocation theory, formal educational institution care reallocates the time spent on family care to institutional care, substituting for the time rural women would have spent on family care (Fitzpatrick, 2010; Dujardin et al., 2018). This relieves the time constraints on rural women’s employment and facilitates better achievement of work-family balance (Heckman, 1974). After addressing the conflict in time allocation between family and work, a comprehensive analysis based on labor market participation theory is needed to determine whether rural women engage in employment (Gronau, 1977). Labor market participation of women is observed only when the wages offered by the market exceed the reserved wages (Heitmueller, 2007). Due to the scarcity of time, family care tends to increase women’s reserved wages and reduce their labor supply (Nakamura and Nakamura, 1992). More expenditures related to family care may reduce their disposable income. More income may increase children’s and elderly’s access to various resources and encourage them to participate in work. Women working outside have two opposing forces (advantages and disadvantages). Therefore, the supply of female labor depends on the net effect of these two opposing forces (Carmichael and Charles, 2003).

### 2.1. Advantages of women’s work

Rural women’s work has a positive impact on child development (Brauner-Otto et al., 2022). On the one hand, increased income can afford more tuition fees (Gong et al., 2015), and enable children to receive better education (Bozick et al., 2010; Nguon, 2012). Women can also improve their social status through work, encouraging their children to pursue a higher level of education (Dhanaraj and Mahambare, 2019). On the other hand, women’s income can increase children’s nutritional intake. Women’s participation in work has a positive effect on weight/height indicators of children aged 12 to 18 months, as the income earned by women can be used to increase expenditures related to children’s food nutrition (Lamontagne et al., 1998). During children’s growth and development, women’s income can increase household dietary diversity by purchasing more food, and compensate for reduced childcare time (Bhalotra, 2010). In addition, when women go out to work and earn wage income, they can provide financial support to the elderly at home (Liu and Wu, 2022). It can significantly improve their life satisfaction, keep the mental health and reduce the degree of depression of the elderly (Cong and Silverstein, 2008).

### 2.2. Disadvantages of women’s work

In contrast to the women’s work-income argument, when women spend more time at work, they spend less time in family care. That causes negative consequences for children and the elderly (Becker, 1965; Sivakami, 2010). When women are busy with work, they may reduce the time they spend with their children. And young children who lack maternal care develop slower than their peers in cognitive and language development, so women have to interrupt their work to take their children’s health development (Boca et al., 2014). When they have more than two young children, the time of women’s work is significantly reduced (Wang et al., 2013), and married women with children less than 6 are less willing to go out to work (Du, 2008). According to the data from the United States, the United Kingdom and Canada, studies have found that caring for the elderly consumes a lot of time and affects women’s willingness to find work (Ettner, 1996; Heitmueller, 2007; Lilly et al., 2010). It is found that caring for the elderly will make women less likely to work, unable to obtain wage income. And they suffer an implicit “wage penalty,” which is an obvious opportunity cost (Kmec, 2011).

### 2.3. Balance

Withdrawing from the labor market is not the only option for women when it comes to caring for their families. Women can balance the family work relationship by choosing positions with more flexible working hours or by self-employment. Flexibility at work means flexibility in working hours and workplace. Flexible work gives women more choice and freedom, and can help them achieve a better work-family balance. Herr and Wolfram (2012) found that women who had chosen flexible employment before having children tended to be more likely to keep their jobs after having children, which partly confirms that more flexible work arrangements help women to balance family and work. Some studies have also found that childcare increases the probability of self-employment for women (Noseleit, 2014; Semykina, 2018), especially for women with children aged 0–3 years (Joona, 2017).

We re-emphasize the positive implications of including women in the labor market, which are integral to securing family benefits (Steiber and Haas, 2012), and are central to women’s quest for gender equality (Brewster and Padavic, 2000; Stier et al., 2012). At the same time, between women’s off-farm employment and family care may be balanced. How to achieve the balance? It requires an analysis of the rural reality that produces this contradiction. Rural areas in China continue to decline (Li et al., 2019), and rural women laborers have to leave their children and the elderly to work in cities. In order to attract them to work nearby, it is necessary to promote the construction of small towns and central villages, and promote the transformation of rural areas from a purely agriculture-based economy to a more diversified economy (Li et al., 2018; Zhang et al., 2019). The developed rural economy creates more employment opportunities (Li et al., 2016), enables the rural population to have better jobs to support their families and maintain a livelihood (Li and Feng, 2021). People are willing to work in their hometowns, which objectively eases the contradiction between family care and going out to work of rural women (Stier and Mandel, 2009).

In conclusion, the effect of family care on women’s work lacks of clear theoretical support. That is often an important empirical issue. However, it is reasonable to assume that women’s proximity to work and access to relevant public services support can alleviate time shortages and reduce the likelihood of dropping out of the labor market (White et al., 2003; Budig et al., 2012).

## 3. Research hypothesis

### 3.1. Theoretical explanation

**(a) Time allocation theory**

Rural women in general face relatively limited employment opportunities, especially considering potential constraints related to working hours and locations. As a result, they must make trade-offs between their family and work responsibilities. This can be primarily analyzed and explained through the lens of time allocation theory. Additionally, this theory examines how rural women allocate their time among various activities to meet diverse needs and goals. According to the theory of time allocation, caring for the family requires rural women to invest a significant amount of time and energy. As time and energy are limited, this squeezes the available work time and reduces the likelihood of employment (Ding et al., 2020). Rural women need to optimize their time allocation between family and work in order to maximize personal and family utility.

**(b) Theory of division of family roles**

The choices rural women make between work and family care also involve the theory of division of family roles. The theory of division of family roles emphasizes society’s expectations for the roles of men and women in both the household and employment contexts. Traditionally, women have been responsible for family caregiving, a division of roles that leads to women investing more time and effort in household care, thereby constraining their opportunities in the job market. According to the theory of division of family roles, men primarily assume the “productive role” and are the main source of family income. Women are expected to take on more family responsibilities, including childbirth, family care, and maintaining the daily operations of the household (Hank and Kreyenfeld, 2003; Sigle-Rushton and Waldfogel, 2007). The theory of division of family roles is widely regarded as rational and efficient in economic terms. It is based on the physical differences and comparative advantages between genders, leading to a natural division (Budig et al., 2012). With the development of society, rural women are also continuously pursuing personal values, such as attaining higher income and advancing to higher positions.

**(c) Social support theory**

However, the time available for them to allocate is limited, and balancing family care and work is challenging. This balance often requires external support, particularly formal care provided by kindergartens or schools, to replace rural women in family care responsibilities (Gelbach, 2002). Social support theory explains how rural women individuals balance the relationship between family caregiving and employment through kindergarten and other social institutions. These institutions can take care of children during working hours, alleviating the burden of women in terms of family caregiving, allowing them more time and energy to invest in their employment. With reliable childcare services, they can more easily engage in full-time work or pursue more challenging careers. Childcare facilities like kindergartens offer flexible services based on family needs, enabling women to arrange childcare according to their work hours and daily schedules, thus achieving a better work-life balance.

It should be noted that when working, an individual cannot simultaneously engage in family activities, thus requiring a consideration of the opportunity costs between work and family. Various theories intermingle, and a thorough analysis reveals that the latter two theories discussed in this paper are all based on the foundation of time allocation theory. As it is commonly known, each person has only “twenty-four hours” in a day.

### 3.2. Support from existing empirical studies

Using micro-level data allows for a more accurate understanding of the relationship between childcare and rural women’s employment. By analyzing the CHNS data from China for the years 1997–2006, the study found that the care of children above 6 significantly impacts rural women’s employment (Yuan, 2010). Using data from the UK’s General Household Survey, it was found that women who engaged in high-intensity childcare work experienced a 10% reduction in their wages (Carmichael and Charles, 1998). Analyzing data from the US Health and Retirement Study, it was discovered that women who took on caregiving responsibilities have 3–10 fewer hours of work per week compared to women who did not have caregiving responsibilities (Houtven et al., 2013). Furthermore, through statistical analysis of data from the European Union Statistics on Income and Living Conditions, it was found that the number of children had a negative impact on the labor force participation of women (Viitanen, 2005). There was also research that utilized longitudinal survey data of young women, which found that women with one child experienced a 4% wage penalty, while women with two or more children experienced a 10% wage penalty (Waldfogel, 1997).

The data reflecting the relationship between rural women’s caregiving responsibilities and work may suffer from endogeneity, leading to biased results and affecting an accurate understanding of the true relationship between caregiving and work. Based on the approaches used to address endogeneity issues, the literature can be categorized into three types. The first type of research treated caregiving as an exogenous variable, disregarding its potential intrinsic linkages with women’s labor force participation and working hours. Under this exogeneity assumption, studies have found a significant negative correlation between caregiving and women’s labor force participation and working hours (Carmichael and Charles, 2003; Lilly et al., 2010). The second type of research employed instrumental variable methods to address endogeneity issues. For example, parental health and mortality were used as instrumental variables to mitigate endogeneity arising from caregiving activities (Heitmueller, 2007; Bolin et al., 2008). The third type of research utilized panel data methods to control for endogeneity resulting from unobserved individual heterogeneity. For instance, simultaneous quantile regression methods with fixed effects or dynamic panel fixed effects models were employed to examine the relationship between caregiving and women’s employment (Budig and Hodges, 2010; Casado et al., 2011).

Improving the market accessibility of caregiving is an important factor in women’s participation in the labor market (Gu, 2020). Basic education public services, by substituting for mothers’ time investment in childcare, helps reconcile the time conflict between caregiving and work. The lack of accessible basic education public services can adversely affect the labor supply of rural women (Heckman, 1974; Fitzpatrick, 2010; Dujardin et al., 2018). Basic education public services enable mothers to re-enter the labor market more quickly after childbirth, ensuring the stability of their employment. The establishment of childcare facilities in communities can encourage women to choose formal caregiving, thereby increasing women’s labor force participation rates (Cascio, 2009; Du and Dong, 2010). As the number of kindergartens increases, it not only enhances women’s labor force participation rates but also increases their weekly working hours (Berlinski and Galiani, 2007).

### 3.3. Interview method

To gain a deeper understanding of rural women’s time allocation between caregiving and employment, an interview method was employed to explore their experiences in family care and employment by interpreting their subjective accounts (Wang, 2017). This approach allowed for the collection of firsthand and intuitive data. The interviews were conducted through online calls, with a total of 12 rural women participating. Each participant’s interview lasted no less than 1 h. The women involved in the interviews were from four cities in Hebei Province and all had rural household registrations. Their ages ranged from 20 to 49 years old, with nine women between the ages of 20 and 30. All interviewees were married and had at least one child, whose ages ranged from 1 to 14 years old. The industries represented included catering, handicrafts, clothing, and domestic services. Compared to agricultural production, these non-agricultural jobs could provide continuous and stable income.

Based on the interview data, all 12 women faced time constraints due to family care responsibilities. Four women were forced to interrupt their work due to caring for their children and elderly family members. Others alleviated the time constraints on their work by choosing different caregiving arrangements. The four women who had interrupted their work emphasized that their husbands believed they made no contribution to the family’s finances and always had to ask their husbands for money. They desired to work outside the home to achieve economic independence and gain respect from their family members. One woman who did not interrupt her work went to work during the day and returned home in the evening to do laundry, cook, and spend time with her family. She not only earned income but also realized her personal value. This woman was satisfied with her life situation. In summary, among the eight women who continued working, other caregiving arrangements were employed. These included: (i) three women chose to send their children to kindergartens or primary schools. (ii) two women had physically healthy elderly relatives at home who provided intergenerational care, allowing them to focus on their work. (iii) three women took their children with them and cared for them while weaving handicrafts in their own village.

### 3.4. Mathematical verification

Married women allocate time between work, leisure, and housework to maximize the total utility of the entire family. Optimization of personal time allocation for rural women is the starting point for studying the impact of family care on labor supply. Under 24-h-a-day conditions, excluding sleep time, personal time allocation refers to the proportion of women’s disposable time spent on work, housework and leisure. Assume that female time allocation mainly includes: daily consumption (*c*), time to take care of children and the elderly (*h*_*g*_), working hours (*h*_*w*_), leisure time (*l*), the price of daily consumption (*P*_*c*_), wage rate per unit of working time (*w*), husband’s wage income (*M*), family’s income from non-labor sources (*F*). Higher-level public services will undoubtedly reduce the employment costs of rural women and alleviate rural women’s worries about going out for employment. This welfare effect will increase working hours (*h*_*t*_). In 1 day, it is assumed that female labor providers must sleep 8 h, and the remaining time at their disposal are 16 h. Then the optimal allocation of female personal time can be expressed as:


(1) $$M\,a\,x\,U=v\,\left(c\right)+u\,\left(l\right)+s\,\left(h_{w}+h_{t}-h_{g}\right)$$

$$s.t.\left\{ \begin{array}{c} P_{c}\cdot c\leq w\,\left(h_{w}+h_{t}-h_{g}\right)+M+F\\ l+h_{w}+h_{t}-h_{g}=16 \end{array}\right.$$

Among them, *v*(⋅), *u*(⋅), and (⋅) are all continuous second-order derivable quasi-concave functions. When preferences are continuous, they can be represented by a utility function. The concave nature of the utility function reflects the law of diminishing marginal utility. This means that the first-order derivative of the utility function is greater than 0; the second-order derivative is less than 0. The strictly concave nature of the objective function ensures the uniqueness of the solution to this optimization problem.

Female labor providers maximize their own utility under the above budget constraints and time constraints. We construct a Lagrangian function:


(2) $$L=v\,\left(c\right)+u\,\left(16+h_{g}-h_{w}-h_{t}\right)+s\,\left(h_{w}+h_{t}-h_{g}\right)$$

$$+\lambda_{1}\,\left[w\,\left(h_{w}+h_{t}-h_{g}\right)+M+F-P_{c}\cdot c\right]$$

$$+\lambda_{2}\,\left(16-l-h_{w}-h_{t}+h_{g}\right)$$

Find the first derivative of *h*_*g*_, *h*_*w*_, and *h*_*t*_ in the above formula and obtain the Kuhn-Tucker condition (one of the most important theoretical results in the field of non-linear programming and is necessary to determine a point as an extremum) according to the aforementioned constraints:


(3) $$\left\{ \begin{array}{c} \frac{\partial L}{\partial h_{g}}=u_{h_{g}}^{\prime }\,\left(\cdot \right)-\frac{\partial s\,\left(\cdot \right)}{\partial h_{g}}-\lambda_{1}\cdot w+\lambda_{2}=0\\ \frac{\partial L}{\partial h_{w}}=-u_{h_{w}}^{\prime }\,\left(\cdot \right)-\frac{\partial s\,\left(\cdot \right)}{\partial h_{w}}+\lambda_{1}\cdot w-\lambda_{2}=0\\ \frac{\partial L}{\partial h_{t}}=-u_{h_{t}}^{\prime }\,\left(\cdot \right)-\frac{\partial s\,\left(\cdot \right)}{\partial h_{t}}+\lambda_{1}\cdot w-\lambda_{2}=0\\ \frac{\partial L}{\partial \lambda_{1}}=w\,\left(h_{w}+h_{t}-h_{g}\right)+M+F-P_{c}\cdot c=0\\ \frac{\partial L}{\partial \lambda_{2}}=16-l-h_{w}-h_{t}+h_{g}=0 \end{array}\right.$$

By shifting the items in (3), the marginal utility of time support provided by family care, work, and public services is obtained as follows:


(4) $$\left\{ \begin{array}{c} \frac{\partial s\,\left(\cdot \right)}{\partial h_{g}}=u_{h_{g}}^{\prime }\,\left(\cdot \right)-\lambda_{1}\cdot w+\lambda_{2}\\ \frac{\partial s\,\left(\cdot \right)}{\partial h_{w}}=u_{h_{w}}^{\prime }\,\left(\cdot \right)-\lambda_{1}\cdot w+\lambda_{2}\\ \frac{\partial s\,\left(\cdot \right)}{\partial h_{t}}=u_{h_{t}}^{\prime }\,\left(\cdot \right)-\lambda_{1}\cdot w+\lambda_{2} \end{array}\right.$$

In order to further obtain the marginal effect of family care and public services on working hours, the second-order derivative of (4) is obtained. The marginal utility of time is as follows:


(5) $$\left\{ \begin{array}{c} \frac{\partial h_{w}}{\partial h_{g}}=\frac{\partial s^{2}/\partial h_{g}^{2}}{\partial s^{2}/\partial h_{g}\cdot \partial h_{w}}=\frac{u_{h_{g}}^{{}^{\prime \prime }}\,\left(\cdot \right)}{-u_{h_{g},h_{w}}^{{}^{\prime \prime }}\,\left(\cdot \right)}<0\\ \frac{\partial h_{w}}{\partial h_{t}}=\frac{\partial s^{2}/\partial h_{t}^{2}}{\partial s^{2}/\partial h_{t}\cdot \partial h_{w}}=\frac{-u_{h_{t}}^{{}^{\prime \prime }}\,\left(\cdot \right)}{-u_{h_{t},h_{w}}^{{}^{\prime \prime }}\,\left(\cdot \right)}>0 \end{array}\right.$$

According to $\frac{\partial h_{w}}{\partial h_{g}}<0$, The increased utility of home care will allow women to increase their care time and reduce working hours. So put forward the research hypothesis:

**Hypothesis 1a:** When the proportion of elderly support in family care is higher, the likelihood of women engaging in employment outside the home is lower.

**Hypothesis 1b:** When the proportion of child support in family care is higher, the likelihood of women engaging in employment outside the home is lower.

Derived from $\frac{\partial h_{w}}{\partial h_{t}}>0$, better public services will increase women’s working hours and reduce family working hours. In rural areas of China, married women need to undertake a lot of housework, which will affect their employment possibilities and working time. Public services play a role in caring for children and the elderly, and alleviate the constraints of housework hours on women’s work. Therefore, the following hypothesis is proposed:

**Hypothesis 2:** Public services provide time support to alleviate the constraints of female family care, thereby increasing the likelihood of women engaging in employment outside the home in rural China.

Based on the above analysis, in theory, taking care of children and the elderly imposes time constraints on rural women’s going out to work, reducing the working hours of rural women. The welfare effect generated as rural public services can adjust these time constraints and encourage rural women to go out to work.

**FIGURE 4:**
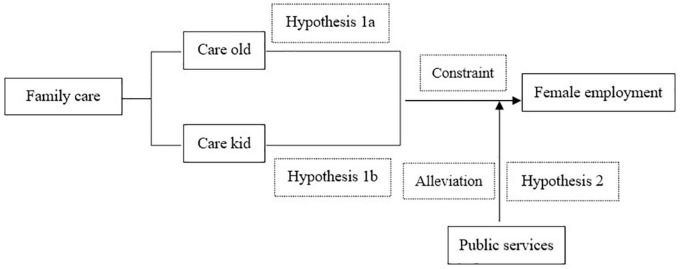
Research framework.

The research framework is shown as Figure 4.

## 4. Research design

### 4.1. Data and descriptive analysis

The data comes from the “China Family Panel Studies” (CFPS) conducted by the Institute of Social Science Survey (ISSS) of Peking University. It is a national, large-scale, multidisciplinary social tracking survey project. The survey includes all family members of sample households in 31 provinces in China. The sample covers a wide range and can be regarded as a nationally representative sample. A total of 2020 data is not yet fully disclosed. The latest year for the available data is 2018. The object of this paper is 3,314 married women aged 20–55 with rural household registration.

Table 1 presents the descriptive results for the explained variables and explanatory variables. We found that 53.3% of rural married female laborers went out to work, about 28% of married women needed to take care of children, and 22.6% of women needed to care the elderly. The proportion of caring for the elderly was slightly lower than the proportion of caring for children. The above data preliminarily shows that part of the married women in rural China who cannot work because they need to take care of children and the elderly.

**TABLE 1:** Descriptive analysis.

Variable	Variable definition	Mean	Sd	Min	Max
Employ	The status of female labor participation	0.533	0.499	0	1
Carekid	The ratio of the number of children aged 0–14 to the total family members in the sample	0.280	0.182	0	3
Careold	The ratio of the number of adults over 60 to the total family members in the sample	0.226	0.235	0	2
Age	Age of female	35.02	7.056	20	54
Edu1_female	Illiteracy or Primary school education of female	0.458	0.498	0	1
Edu2_female	Junior high school education of female	0.321	0.467	0	1
Edu3_female	High school education of female	0.125	0.331	0	1
Edu4_female	Bachelor degree or above of female	0.0960	0.295	0	1
Health	Health level of female	0.770	0.421	0	1
Ecoadmin	Satisfaction with her husband’s economic contribution	4.194	0.971	1	5
Localincome	Local ranking of income level	2.532	0.952	1	5
Age_male	Age of husband	37.05	7.103	18	76
Edu1_male	Illiteracy or Primary school education of husband	0.387	0.487	0	1
Edu2_male	Junior high school education of husband	0.168	0.374	0	1
Edu3_male	High school education of husband	0.140	0.347	0	1
Edu4_male	Bachelor degree or above of husband	0.110	0.313	0	1
Consume	Total household consumption	3496	4261	0	20000
Outcome	Expenditure to maintain social relations	3631	4074	200	20000
Income_past	Total income in the past year	52268	39662	4000	180000
Family_num	Number of family members	5.066	1.854	1	17
Income_per	Per capita household income	11503	9798	340	44000
Distance_county	Distance to the county	47.98	40.31	0	280
Distance_province	Distance to the provincial capital	558.0	749.6	0	6400
East	Eastern region	0.355	0.479	0	1
Midland	Central region	0.361	0.481	0	1
West	Western region	0.284	0.451	0	1

^a^The result is calculated by the author.

^b^In rural China, women’s employment is more flexible. Although the article wants to explore the nature of rural women’s employment, there is a lack of statistical information on cross-regional employment for more than 6 months and part-time employment close to home. Therefore, the definition of the explanatory variable in the article is based on the questionnaire “Is your current job agricultural or non-agricultural work.” This is also the common practice adopted in most of the articles in the absence of relevant statistical information.

It was noticeable that *Edu_female* indicated the level of education of women. It was divided into *Edu1-Edu4* according to different education stages, such as 6 years of primary school, 9 years of junior high school, 12 years of high school, and 16 years of university and above. It showed that women’s education level was primary school and below, junior high school, high school and university level or above. The definitions and interpretation of other variables are shown in Table 1.

Descriptive analysis provided statistical results on the relationship between family care and female employment. In order to verify the causal relationship between caregiving and female employment, we will employ different methods and analytical techniques in the empirical analysis section to determine the actual impact of caregiving on female labor force participation. First, we included more control variables in the model using a stepwise regression approach to control for potential confounding factors. Second, we employed propensity score matching method to address sample selection bias. Third, since there is a bidirectional causal relationship between caregiving and female employment, we used instrumental variable estimation to test for endogeneity. Last, we examined the robustness of the results by replacing explanatory variables and using alternative model specifications.

### 4.2. Model setting

The model of family care activities for rural married women’s employment as follows:


(6) $$Employ=f(\beta +\alpha_{1}Care_{i}+\alpha_{2}X_{m\,i}+\alpha_{3}X_{f\,i}$$

$$+\alpha_{4}X_{h\,i}+\alpha_{5}X_{r\,i}+\alpha_{6}X_{d\,i}+\varepsilon_{i})$$

The explained variable *Employ* is the status of female labor participation. If they are engaged in non-agricultural work, the value is 1, otherwise it is 0. *Care*_*i*_ represents family care, including old-age dependency ratio (the ratio of the number of adults over 60 to the total family members in the sample), and the child dependency ratio (the ratio of the number of children aged 0–14 to the total family members in the sample). *X*_*mi*_ is female individual characteristic variable, including *Age, Edu_female, Health, Ecoadmin*, and *Localincome*. *X*_*fi*_ is husband’s individual characteristic variable, including *Age_male, Edu_male. X*_*hi*_ is family characteristic variable, including *Consume*, *Outcome*, *Income_past*, *Family_num*, *Income_per*. *X*_*ri*_ is village characteristic variable, including *Distance_county*, *Distance_province*. *X*_*di*_ is regional characteristic variable, including *East*, *Midland*, *West*, *i* represents different individuals. Hence, women’s decision to go out to work is a function *f*(⋅) of family care, individual female characteristics, husband characteristics, family status, village characteristics, and regional characteristics.

For the dichotomous results, the models that can be used for parameter estimation are probit, logit and linear probability model. We chose logit model for parameter estimation based on the following reasons. First, Logit model is widely used as a discrete choice model. It is a common method for empirical analysis in sociology and econometrics. Second, the Logit model yields more accurate and stable results compared to the linear probability model. It provides interpretable numerical estimates that represent probabilities. Third, the Logit model offers advantages in predicting probabilities, allowing for an examination of how these estimates vary under different conditions. It assumes that the random perturbation terms follow a logistic distribution and provides analytic expressions. Fourth, the disadvantage of probit is that it has to do multiple integrations, resulting in less efficient operations. However, each model estimation method has its own advantages and disadvantages. It is important to objectively acknowledge the limitations associated with the Logit model. Specifically, the Logit model does not account for parameter heterogeneity. Here is the average marginal effect and its expression is shown in equation (7).


(7) P(Employ=1|x)=F(x,β)=

$$\frac{\exp\left(\beta +\alpha_{1}\,C\,a\,r\,e_{i}+\alpha_{2}\,X_{m\,i}+\alpha_{3}\,X_{f\,i}+\alpha_{4}\,X_{h\,i}+\alpha_{5}\,X_{r\,i}+\alpha_{6}\,X_{d\,i}\right)}{1+\exp\left(\beta +\alpha_{1}\,C\,a\,r\,e_{i}+\alpha_{2}\,X_{m\,i}+\alpha_{3}\,X_{f\,i}+\alpha_{4}\,X_{h\,i}+\alpha_{5}\,X_{r\,i}+\alpha_{6}\,X_{d\,i}\right)}$$

$$+\varepsilon_{i}$$

*P* represents the probability of whether a woman goes out to work.

## 5. Results and discussion

### 5.1. Basic regression

(1) to (6) columns in Table 2 represented the following Logit regression results. Column (1) was the regression results without considering any control variables. Column (2) only controlled for relevant variables related to women’s individual characteristics. Column (3) was the regression results simultaneously controlling for women’s individual characteristics and their husbands’ individual characteristics. Column (4) was the regression results simultaneously controlling for women’s individual characteristics, their husbands’ individual characteristics, and family level characteristics. Column (5) was the regression results simultaneously controlling for women’s individual characteristics, their husbands’ individual characteristics, family level characteristics, and village-level characteristics. Column (6) was the regression results simultaneously controlling for women’s individual characteristics, their husbands’ individual characteristics, family characteristics, village characteristics, and regional characteristics. Based on sociological theories and economic demand theories, column (6) comprehensively considered both rural women’s own characteristics and the economic and social conditions. Therefore, we analyzed the regression results in this column for further insights. Considering that more village information was used, all standard errors were clustered at the village level.

**TABLE 2:** Regression results.

Variable	(1)	(2)	(3)	(4)	(5)	(6)
Carekid	−0.2247*** (0.0614)	−0.2016*** (0.0535)	−0.1799*** (0.0488)	−0.2367*** (0.0665)	−0.1883*** (0.0719)	−0.1727** (0.0766)
Careold	0.0047 (0.0420)	0.1711*** (0.0436)	0.1607*** (0.0420)	0.0440 (0.0625)	0.0474 (0.0616)	0.0231 (0.0616)
Age		0.0126 (0.0113)	0.0236* (0.0139)	0.0100 (0.0176)	0.0127 (0.0197)	0.0013 (0.0196)
Age 2		−0.0003* (0.0002)	−0.0004** (0.0002)	−0.0002 (0.0003)	−0.0002 (0.0003)	−0.0001 (0.0003)
Edu1_female		−0.7213*** (0.0536)	−0.6383*** (0.0536)	−0.3053*** (0.0675)	−0.2312*** (0.0621)	−0.2013*** (0.0633)
Edu2_female		−0.5073*** (0.0554)	−0.4766*** (0.0549)	−0.2016*** (0.0676)	−0.1368** (0.0620)	−0.1205* (0.0634)
Edu3_female		−0.2836*** (0.0648)	−0.2890*** (0.0635)	−0.1160 (0.0771)	−0.0247 (0.0729)	−0.0193 (0.0735)
Health		0.0230 (0.0185)	0.0261 (0.0186)	0.0151 (0.0241)	0.0064 (0.0245)	0.0036 (0.0256)
Ecoadmin		−0.0304*** (0.0079)	−0.0316*** (0.0079)	−0.0264** (0.0117)	−0.0185 (0.0126)	−0.0207* (0.0125)
Localincome		−0.0032 (0.0087)	−0.0033 (0.0085)	−0.0195* (0.0117)	−0.0254** (0.0118)	−0.0219* (0.0121)
Age_male			−0.0133 (0.0128)	−0.0343** (0.0149)	−0.0343* (0.0189)	−0.0183 (0.0190)
Age_male2			0.0001 (0.0002)	0.0003* (0.0002)	0.0003 (0.0003)	0.0002 (0.0003)
Edu1_male			−0.0766*** (0.0211)	−0.0562** (0.0251)	−0.0604** (0.0260)	−0.0672*** (0.0257)
Edu2_male			0.0954*** (0.0295)	0.0065 (0.0351)	0.0085 (0.0388)	−0.0246 (0.0505)
Edu3_male			0.1415*** (0.0287)	0.0198 (0.0402)	−0.0007 (0.0426)	−0.0155 (0.0435)
Lnconsume				−0.0158 (0.0098)	−0.0081 (0.0104)	0.0015 (0.0104)
Lnoutcome				−0.0086 (0.0102)	−0.0060 (0.0107)	0.0004 (0.0106)
Lnincome_past				−0.0067 (0.0183)	−0.0167 (0.0190)	−0.0243 (0.0198)
Lnincome_per				0.1121*** (0.0147)	0.1074*** (0.0155)	0.1071*** (0.0160)
Family_num				0.0141** (0.0072)	0.0151** (0.0074)	0.0144** (0.0071)
Lndistance_county					−0.0385** (0.0152)	−0.0423*** (0.0155)
Lndistance_province					−0.0333** (0.0148)	−0.0222 (0.0152)
East						0.1498*** (0.0388)
Midland						0.0840** (0.0355)
*N*	3313	3099	3043	1403	1255	1210
Pseudo *R*^2^	0.004	0.198	0.221	0.234	0.262	0.270

Source: The results are calculated by the author. ^a“^*Lnconsume*” *is logarithmic to* “*consume*,” *and* “*Ln*” *precedes other variables with the same meaning*. For variables taking logarithms are mainly based on the following considerations: first, generally in economic models, the estimated coefficients can be interpreted as elasticities; second, the degree of sample heteroskedasticity can be reduced; and finally, the volatility of the variables is reduced to match the level of volatility of other variables. ^b^Table shows the results of stepwise regression method. To verify the relationship between the core variables, the other variables were strictly controlled. ^c^All standard errors are clustered at the village level.

*p < 0.10, **p < 0.05, ***p < 0.01.

The coefficient of the first key explanatory variable *Careold* was positive. Most columns did not pass the significance tests. Married women who took care of the elderly on their own increased the probability of working outside by 2.31–17.11%. This result indicated that taking care of the elderly did not hinder rural women’s employment. The regression results were inconsistent with Hypothesis 1a. The reason is that most of the individuals aged 60–70 are included in the sample. Sociological theories explain the seemingly contradictory relationship between taking care of the elderly and rural women’s employment from the perspectives of elderly public services and social support. As modern society gradually provides more infrastructure and social support to address the issue of elderly care, such as nursing homes and day care centers, these facilities reduce the caregiving burden on women in their households. Meanwhile, the health level of the elderly in this age group is acceptable and can help young women share the responsibilities of childcare and housework, which encourages them to engage in higher-paid non-agricultural work. This relates to the family role reshaping, where the individuals requiring care can sometimes transition into roles of caring for other family members. This reshaping of roles can alleviate the burden on rural women and provide them with more time and energy to participate in employment activities.

The coefficient of the second key explanatory variable *Carekid* was negative. The results of each column have passed significance tests at a minimum level of 5%. Taking care of children reduced the probability of married women working outside by 17.27–23.67%. Hypothesis 1b was confirmed. In comparison to taking care of the elderly, it was evident that childcare had a greater restrictive effect on married women in rural areas when it came to pursuing work opportunities. In China, rural households often rely on the income of male to sustain their livelihood. Due to a lack of other caregiving resources and social welfare, rural women may be forced to give up work or reduce their working hours. In particular, according to the theory of division of family roles, caregiving responsibilities still disproportionately fall on rural women. This unequal distribution limits women’s opportunities for career development and employment.

With Hypothesis 1b being verified, the article will further explore the impact of the number of children in need of care on women’s work. Additionally, we will examine the role of public service institutions such as kindergartens and primary schools in rural areas in alleviating the conflict between married women’s work and their role in caring for children.

### 5.2. Dealing with sample selection bias

Since different female individuals have varying attitudes toward childcare, such as “forced care” and “active care,” whether or not to take care of children introduces a self-selection problem in the sample. This self-selection problem leads to endogeneity issues, resulting in deviations in the regression results of the model. Propensity score matching (PSM) addresses the issue of selection bias. The propensity score matching method uses the propensity score values to identify one or more individuals from the control group with similar background characteristics as the treatment group for each individual in the treatment group. This minimizes the interference of other confounding factors. Therefore, the PSM method can be used to calculate the net effect of women taking care of children and its impact on their employment status. We divided the sample into two groups: the treatment group consisting of individuals who need to provide family care, and the control group consisting of individuals who do not need to provide family care. To accurately assess the effect of family care on women’s employment, it is essential to control for sample selection bias. The fundamental concept of this method is that by identifying a control group that closely resembles the treatment group, the sample selection bias can be effectively mitigated when assessing the impact of a specific behavior. However, finding a satisfactory match based on a single characteristic alone is challenging, and technical difficulties arise when attempting to match based on multiple characteristics. PSM can consolidate multiple characteristics into one indicator, namely the propensity score. In this way, when estimating the difference between the treatment group and the control group, the noticeable deviations caused by observable characteristics can be eliminated, and the average treatment effect (*ATT*) that influences women’s employment status can be calculated. As shown in Equation (8), the formula is as follows:


(8) $$ATT=E\left[\left(Y_{1\,i}-Y_{0\,i}\right)|D_{i}=1\right]=E\left\{E\left[\left(Y_{1\,i}-Y_{0\,i}\right)|D_{i}=1\right],P\left(X_{i}\right)\right\}$$

$$E\{E\left[Y_{1\,i}|D_{i}=1,P\left(X_{i}\right)\right]-E\left\{E\left[Y_{0\,i}|D_{i}=0,P\left(X_{i}\right)\right]|D_{i}=1\right\}$$

In the above formula, the binary dummy variable *D* represents the childcare constraint, *Y*_1*i*_ and *Y*_0*i*_ are the results of the treatment group and the control group, respectively.

First, propensity score matching is performed on married women with and without care constraints. Female individual characteristics, husband individual characteristics, family characteristics, village residence, and regional characteristics are selected to establish the Logit model. Based on the regression results of the model, the influence on women’s propensity to work outside the home is estimated. Common matching methods include nearest neighbor matching, radius matching, and kernel matching. Selecting different matching methods can verify the robustness of the results from multiple perspectives. Nearest neighbor matching selects the closest control group sample in terms of propensity scores for each sample in the treatment group, resulting in smaller deviations. Radius matching involves setting a predetermined radius, and samples with propensity score differences between the treatment and control groups smaller than the radius are selected as matching samples. Kernel matching assigns different weights to control group individuals based on the proximity of their propensity score values to the propensity score value of the treatment group individuals. This method utilizes all control group samples when the sample size is not large enough, ensuring statistical efficiency. Table 3 shows the proportions and differences (*ATT*) of women’s employment impact between the treatment and control groups, standard deviations, *T*-values, sample sizes of the treatment and control groups. The results obtained by the three methods are similar, indicating robust estimations.

**TABLE 3:** The results of propensity score matching.

Matching method	Treatment group	Control group	*ATT*	Standard deviation	*T*-value	Samples in treatment group	Samples in control group
Nearest neighbor matching (1:1)	0.1882	0.3469	−0.1587	0.0398	−4.39	542	662
Nearest neighbor matching (1:4)	0.1882	0.3344	−0.1462	0.0322	−5.05	542	662
Radius matching	0.1899	0.3421	−0.1522	0.0256	−5.65	537	662
Kernel matching	0.1901	0.3509	−0.1608	0.0232	−6.09	547	663

Source: The results are calculated by the author.

^a^The propensity score required for sample matching satisfies the balance condition.

^b^The standard deviation of the *T*-value is calculated by the Bootstrap method.

^c^The nearest neighbor matching adopts a replacement method.

^d^In the radius matching, the radius is selected as 0.01.

Taking 1:1 nearest neighbor matching as an example, the results of the *ATT* test were described in detail. The average employment level of rural women in the treatment group was 0.1882, while the average employment level of rural women in the control group was 0.3469. The *ATT* value was −0.1587, indicating a negative effect of the treatment group relative to the control group. This difference was statistically significant. It suggested that family care had a negative impact on women’s employment, and women in the treatment group may face time constraints on employment due to increased caregiving responsibilities. The coefficients fluctuated between −16.08 and −14.62%. Under the assumption of controlling for other variables, the probability of women without caregiving constraints working outside the home was significantly higher than that of women with caregiving constraints. The reason may be that, on one hand, women generally bear more caregiving responsibilities, which greatly limits their job opportunities, leading them to leave the workforce to take care of their families. On the other hand, the difficulty of balancing work and family prevents women from participating in the labor market.

**FIGURE 5:**
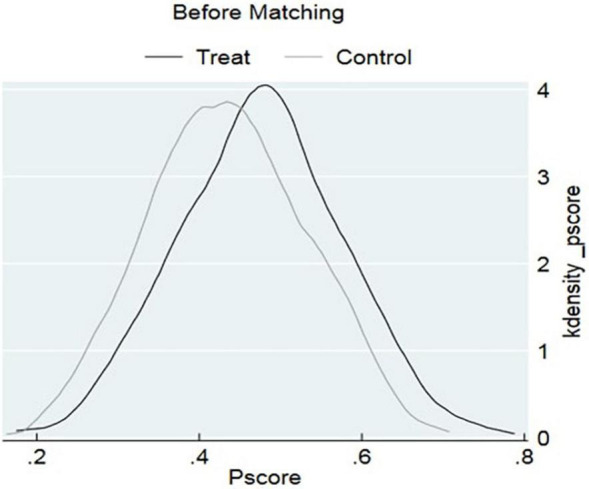
Pscore before matching.

**FIGURE 6:**
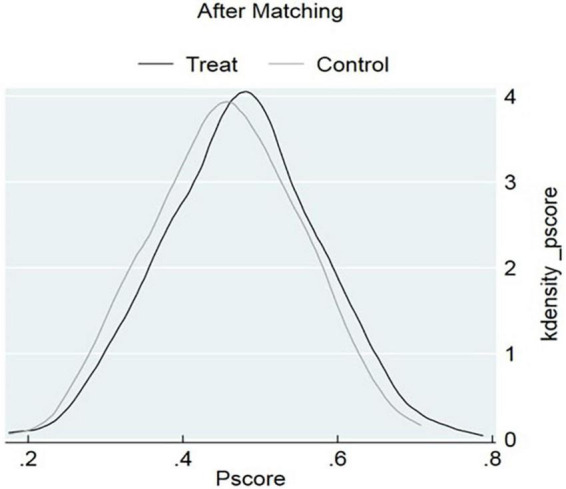
Pscore after matching.

In addition, we generate kernel density function plots to intuitively reflect the differences between the scores before and after matching. The common support region is a fundamental test for assessing the effectiveness of propensity score matching. When the common support domain is large, the probability of sample loss during matching becomes small. By comparing the curves in Figures 5, 6, it was evident that before matching, although there was some overlap between the control and treatment groups, the alignment of the function contours was not high. However, after matching, the overlapping area between the two groups significantly increased, indicating a higher degree of alignment. The propensity scores of the two sample groups became closer. Figure 6 demonstrated that there was a large overlapping interval between the propensity scores of women without childcare constraints and those with childcare constraints. This indicated that most observed values were in the similar range of values, resulting in fewer lost matches and improved representativeness of the samples.

### 5.3. Endogeneity treatment

Regression analysis of the effect of family care on labor participation of rural married women suffers from endogeneity issues due to bidirectional causality. On one hand, childcare activities reduce women’s time investment in work, thereby lowering labor participation. On the other hand, women’s employment also affects their childcare behavior. When women participate in the workforce and earn wages, they may choose to purchase childcare services in the market or rely on other household members to take care of their children. The presence of endogeneity introduces bias in the estimation of regression coefficients. To mitigate this bias, it is necessary to control for endogeneity, and instrumental variable methods are commonly employed to address endogeneity issues. Therefore, this paper adopted an instrumental variable approach to address the endogeneity problem arising from reverse causality. Regarding the selection of instrumental variables related to childcare, previous literature often used information related to grandparents, such as whether grandparents are alive and their health conditions. However, these instrumental variables lack randomness and may lead to a loss of a significant number of samples. In this paper, the variable “duration of living with mothers in the past 12 months” was used as the instrumental variable. Effective instrumental variables need to satisfy two conditions: relevance and exogeneity. It directly affected the probability of women personally taking care of their children, thereby influencing their employment status. Moreover, this instrumental variable did not exhibit significant correlation with other factors that may affect women’s employment. The Wald test results indicated a *p*-value of 0.0277, which suggests the presence of endogeneity at a 5% significance level. The regression coefficient was negative and statistically significant at the 1% level, implying that an increase in childcare had a negative impact on women’s employment. Specifically, the probability of labor participation among rural married women decreased. The results are presented in Table 4.

**TABLE 4:** Endogeneity treatment.

Variable	Carekid	Employ
Carekid	–	−5.4553*** (0.0465)
Live	−0.0027 (0.0032)	
Individual characteristics of women	Yes	Yes
Individual characteristics of husband	Yes	Yes
Family characteristics	Yes	Yes
Village characteristics	Yes	Yes
Regional characteristics	Yes	Yes
*N*	1210	1210

The results are calculated by the author.

*p < 0.10, **p < 0.05, ***p < 0.01.

### 5.4. Robustness test

To verify the robustness of the results, we examined the impact of childcare on women’s employment by replacing explanatory variables and using different models. The results are shown in Table 5. In the first column, the explanatory variable was replaced with “whether cared by the mother.” If the answer was yes, it was assigned a value of 1; otherwise, it was assigned 0. The estimated coefficient was −0.1531, which was statistically significant at the 1% level. This indicated that when children were cared for by their mothers themselves, the probability of women’s employment decreased by 15.31%. Mothers took on more responsibility for childcare, thereby reducing the likelihood of labor participation. In the second column, the explanatory variable was replaced with “number of children in the household,” and an OLS regression was applied. The estimated coefficient was −0.0254, which was statistically significant at the 10% level. As the number of children in the household increased, the probability of women’s employment slightly decreased. This may be because an increase in the number of children in the household added to the caregiving workload, making it difficult for women to balance family and work. In the third column, an OLS regression was used, and the estimated coefficient was −0.1280, which was statistically significant at the 1% level. The estimation results indicated a negative relationship between an increase in childcare and women’s employment. In the fourth column, a Probit model was used, and the estimated coefficient was −0.1705, which was statistically significant at the 5% level. Once again, this confirmed that as childcare increased, the probability of women’s employment decreased. Considering the results from both the replacement of explanatory variables and the transformation of models, the negative impact of childcare on women’s employment was robust.

**TABLE 5:** Robustness test.

Variable	(1)	(2)	(3)	(4)
Carekid	−0.1531*** (0.0207)	−0.0254* (0.0144)	−0.1280*** (0.0420)	−0.1705** (0.0728)
Individual characteristics of women	Yes	Yes	Yes	Yes
Individual characteristics of husband	Yes	Yes	Yes	Yes
Family characteristics	Yes	Yes	Yes	Yes
Village characteristics	Yes	Yes	Yes	Yes
Regional characteristics	Yes	Yes	Yes	Yes
*N*	1211	1211	1210	1210
*R*^2^ or Pseudo *R*^2^	0.298	0.284	0.287	0.271

The results are calculated by the author. All standard errors are clustered at the village level.

*p < 0.10, **p < 0.05, ***p < 0.01.

## 6. Heterogeneity analysis and expanded analysis

### 6.1. Heterogeneity analysis based on women’s age

Grouping the samples based on women’s age, we divided them into three categories: young married women aged 20 to 30, young married women aged 31 to 40, and middle-aged married women aged 41 to 55. The results are presented in Table 6.

**TABLE 6:** Regression of different age.

	(1) Aged 20–30	(2) Aged 31–40	(3) Aged 41–55
Carekid	−0.2636* (0.1485)	−0.2182 (0.1405)	−0.0520 (0.1241)
Individual characteristics of women	Yes	Yes	Yes
Individual characteristics of husband	Yes	Yes	Yes
Family characteristics	Yes	Yes	Yes
Village characteristics	Yes	Yes	Yes
Regional characteristics	Yes	Yes	Yes
*N*	356	535	319
Pseudo *R*^2^	0.292	0.210	0.284

The results are calculated by the author. All standard errors are clustered at the village level.

*p < 0.10, **p < 0.05, ***p < 0.01.

Column (1) showed that childcare had the largest negative impact on the employment of women aged 20 to 30, with a decrease in the probability of engaging in employment by 26.36%. This result can be explained by various theories. Considering the reproductive stage, the age range of 20 to 30 is the peak period of women’s reproductive ability, and many women become mothers during this stage. These women have a more urgent responsibility for childcare, as they may require more time and energy to take care of infants and young children. It can have a significant impact on their work. Furthermore, analyzing from the perspective of career development stage, the age range of 20 to 30 is typically a crucial foundation stage for individual career development. Many women in this stage need to invest more effort in pursuing professional skill training and career advancement. Childcare responsibilities can significantly disrupt their career development because they need to balance work and family responsibilities, which may lead to reduced job opportunities or limitations on working hours. Compared to other age groups, women aged 20 to 30 may face more challenges in independently caring for their children. In young couples at this stage, the husband needs to focus on work to generate income for the family to maintain normal household expenses. This further impacts the employment of rural women.

Column (2) revealed that childcare also had a significant inhibiting effect on the employment of women aged 31 to 40, with a 21.82% decrease in the probability of engaging in employment. Sociological theories suggest that the strong inhibiting effect of childcare on the employment of women in this age group is influenced by traditional gender roles and societal expectations. According to this theory, women in this age range are typically at a crossroads between career and family responsibilities, facing the challenge of balancing work and family. Additionally, the lack of flexible work arrangements and supportive family policies further reinforce the inhibiting effect of childcare on the employment of women aged 31 to 40. Considering the theory of division of family roles, married women in this age group might experience limited family support. For instance, their spouses may still be in a busy work stage and unable to share childcare responsibilities, which fails to alleviate their burdens and create more job possibilities for them.

Column (3) showed that childcare had the weakest inhibitory effect on the employment of women aged 41–55, reducing their probability of going out to work by 5.2%. Compared to women aged 20–30 and 31–40, they have completed the reproductive stage or their children have grown up, thus having lower responsibilities in childcare. As women age, those aged 41–55 may have more confidence and ability to balance work and family responsibilities since their children are capable of taking care of themselves better. The theory of division of family roles suggests that societal expectations of women’s roles may have changed during this age range. Traditional gender role norms may no longer be as strong, and society is more accepting of the professional development of women in this age group. This change in social environment helps alleviate the inhibitory effect of childcare on the employment of women aged 41–55. Rural women in this stage may have accumulated more experience and professional skills in the professional field, which contributes to their competence in their work.

### 6.2. Heterogeneity analysis based on number of children

The impact of childcare on rural married women’s employment may vary depending on the number of children (Houtven et al., 2013). Therefore, we divided the sample into two groups based on the median number of young children: one group with a number of children greater than the median, and the other group with a number of children less than the median. We then performed a Logit regression analysis on each group. The results are presented in Table 7.

**TABLE 7:** Regression of the number of children.

	(1) More	(2) Less
Carekid	−0.3280* (0.1886)	−0.0880 (0.1533)
Individual characteristics of women	Yes	Yes
Individual characteristics of husband	Yes	Yes
Family characteristics	Yes	Yes
Village characteristics	Yes	Yes
Regional characteristics	Yes	Yes
*N*	496	714
Pseudo *R*^2^	0.243	0.336

The results are calculated by the author. All standard errors are clustered at the village level.

*p < 0.10, **p < 0.05, ***p < 0.01.

For households with a number of young children greater than the median, childcare had a significant impact on the employment of rural married women, reducing their probability of going out to work by 32.8%. Family structure theory suggests that as the number of children increases, rural women typically need to invest more time and energy in taking care of their children. This includes childcare, nurturing, maintaining their health, and providing education. These family responsibilities create pressure on women’s time and energy, making it challenging for them to engage in paid work simultaneously. According to the opportunity cost, when the number of children is high, the cost of childcare services can be substantial, sometimes exceeding the income from going out to work. In such cases, women may be willing to sacrifice their time for paid work to personally care for their children. In reality, it has limited educational and employment opportunities in rural areas, and an increase in the number of children may further restrict women’s prospects for employment.

For households with a number of young children less than the median, specifically when there was only one child, childcare reduced the probability of rural women’s employment by 8.8%. However, the result was not statistically significant, indicating that childcare had a weak inhibitory effect on married women’s work outside the home. When rural women have only one child, the caregiving responsibilities are relatively light, meaning they can more easily arrange their time for childcare and have more leisure time to engage in work. Additionally, having only one child results in lower childcare costs, which the family can afford. They can focus on their work without worries, earning income that surpasses the opportunity cost of childcare. These factors make rural women with fewer children more inclined to choose employment.

### 6.3. Heterogeneity analysis based on family income level

To analyze the impact of child caregiving on rural women’s employment under different household income levels, we utilized the quantile grouping function of the Stata software. This function was used to divide the household income variables in the sample into three groups: low-income, middle-income, and high-income, as defined in Table 8. It is important to note that the household income thresholds used for classification were not based on government-published data but were determined by the authors based on the actual average income of rural Chinese families in the sample. The cutoff points for the groups were automatically calculated by the statistical software. The quantile points for annual income were set at 30,100 CNY and 60,400 CNY per family, indicating that families with incomes below 30,100 CNY were classified as low-income, while those with incomes above 60,400 CNY were classified as high-income. The range between these two values represented middle-income households.

**TABLE 8:** Analysis of the interaction mechanism between income level and childcare.

	(1) Rural low income	(2) Rural middle income	(3) Rural high income
Carekid	−0.1052 (0.0802)	−0.1610* (0.0897)	−0.2087** (0.0954)
Jiaohu1_1	−0.1840* (0.1026)		
Jiaohu1_2		0.0047 (0.0756)	
Jiaohu1_3			0.1875* (0.1033)
Individual characteristics of women	Yes	Yes	Yes
Individual characteristics of husband	Yes	Yes	Yes
Family characteristics	Yes	Yes	Yes
Village characteristics	Yes	Yes	Yes
Regional characteristics	Yes	Yes	Yes
*N*	991	991	991
Pseudo *R*^2^	0.270	0.268	0.270

The results are calculated by the author. All standard errors are clustered at the village level.

*p < 0.10, **p < 0.05, ***p < 0.01.

The economic conditions of rural households may be limited, and bearing the additional cost of childcare could impose a significant financial burden on families. Therefore, the income level of the household plays a crucial role in determining the decision of rural women to participate in work or engage in childcare. The impact of childcare on women’s workforce participation in rural areas was examined by considering the intersection of childcare service variables and childcare variables. Column (1) revealed that, for low-income families, childcare significantly decreased the likelihood of rural women working outside the home by 18.40%. This is due to their inability to afford childcare services and alleviate their burden. In column (2), it indicated that women from middle-income families in rural areas could utilize childcare institutions to care for their children, which encouraged them to work outside the home, albeit to a lesser extent, with a probability of 0.47%. Column (3) demonstrated that, for high-income rural families, women faced no financial constraints and possessed sufficient income to cover childcare expenses. As a result, they were more inclined to engage in work outside the home, with a probability of 18.75%.

It is evident that there is a clear positive relationship between childcare services and family income. The impact of childcare on rural women’s employment involves multiple aspects, including human capital, family responsibilities, gender roles, and financial factors. These intertwined factors collectively influence the status and income levels of rural women in the job market. Governments and societies can assist in mitigating the impact of childcare on rural women’s employment by improving kindergarten facilities, implementing gender equality policies, and providing economic support.

### 6.4. Extended analysis: public services and women’s employment

We established different sample groups to examine the role of public services, such as kindergartens and primary schools, in alleviating the childcare burden faced by married women. The results are presented in Table 9.

**TABLE 9:** The role of public services.

	(1) Kindergarten	(2) Primary schools	(3) Interaction effect 1	(4) Interaction effect 2
Carekid	−0.3508** (0.1538)	−0.0433 (0.1091)	−0.1395* (0.0773)	−0.0161 (0.0764)
Jiaohu2_1	0.0397 (0.0529)		0.0187 (0.0751)	
Jiaohu2_2		−0.1195* (0.0699)		−0.0646 (0.0552)
Individual characteristics of women	Yes	Yes	Yes	Yes
Individual characteristics of husband	Yes	Yes	Yes	Yes
Family characteristics	Yes	Yes	Yes	Yes
Village characteristics	Yes	Yes	Yes	Yes
Regional characteristics	Yes	Yes	Yes	Yes
*N*	506	784	506	784
*R*^2^ or Pseudo *R*^2^	0.312	0.205	0.333	0.193

The results are calculated by the author. All standard errors are clustered at the village level.

*p < 0.10, **p < 0.05, ***p < 0.01.

In rural areas, women often encounter difficulties in finding someone to take care of their children while pursuing job opportunities. Kindergartens and primary schools provide a safe, healthy, and educational care environment, enabling rural women to entrust their children to schools and teachers. This helps alleviate the time constraints they face while working. Column (1) demonstrated that the presence of kindergartens in the village increased the probability of female employment by 3.97%. Conversely, when there were no kindergartens, the probability of hindering women from going out to work was −35.08%. Thus, Hypothesis 2 is partially confirmed. However, column (2) revealed that the inhibitory effect of public services such as primary schools on women’s employment was −11.95%. In contrast, when there were no primary schools, the inhibitory effect was only −4.33%.

To further validate the robustness of the aforementioned findings, OLS estimates were employed to test the aforementioned inferences in column (3) and column (4). The regression results for the interaction term between the presence of a kindergarten and childcare indicated a 1.87% increase in the probability of rural women’s employment (when there were kindergartens) and a 13.95% decrease (when there were no kindergartens). This once again confirmed that public services such as kindergartens could alleviate the conflict faced by rural married women between childcare responsibilities and seeking employment opportunities. Regarding the interaction between primary schools and childcare in the village, the results revealed a 6.46% reduction in the probability of rural women’s employment (when there were primary schools) and a 1.61% reduction (when there were no primary schools). This demonstrated that the constraint on working outside the home due to the care of primary school-aged children was further reinforced.

According to social support theory, the establishment of kindergartens provides professional care services for rural women, alleviating the time constraints they face and providing them with the opportunity to participate in work. The empirical results demonstrate that kindergartens can assist married women in childcare and promote their employment. However, we were surprised to find that even when primary education facilities are available in the village, they fail to encourage greater female participation in the workforce. The theory of division of family roles offers an explanation for this contradiction. This theory posits that mothers, as the primary caregivers for their children, bear the responsibility of caring for and educating them. Particularly during the primary school stage, mothers play a vital role in their children’s learning and development, being regarded as their “first teachers.” Therefore, mothers may face conflicting choices: fulfilling their maternal duties while pursuing employment opportunities and career development. The results indicate their inclination to prioritize their role as mothers, as they forgo employment opportunities. This suggests that they believe the presence of mothers during the primary school stage is crucial for their children’s learning progress and psychological wellbeing.

## 7. Discussion

Sociological theories and practices provide some useful explanations for rural women’s employment. In rural areas, the concept of gender role division is deeply ingrained. It emphasizes women’s role in household care, which may restrict the opportunities for rural women to obtain employment. The theory of division of family roles also contributes to explaining rural women’s labor participation (Vlasblom and Schippers, 2004). By studying the impact of family care on women’s employment, it reveals the constraining factors of female labor participation. This helps deepen our understanding of female participation decision-making and employment opportunities. Upon applying the social support theory, this paper examines the substitution effects of public education services on female caregiving, emphasizing the role of public services in promoting women’s employment. By empirically investigating these relationships, this paper provides valuable insights into the factors influencing rural women’s employment and highlights the potential role of public services in alleviating the burden of care and facilitating women’s participation in the workforce.

(a) Constructing a Logit model to analyze the impact of elderly care on employment of married women in rural areas, it was found that caring for the elderly did not impose constraints on female employment. The conclusion is not consistent with existing typical studies (Bolin et al., 2008; Huang, 2012). According to the intergenerational support, when elderly individuals are in good health, they transition from being the recipients of care to becoming caregivers for their family members. In order to help rural women balance their family and work responsibilities, they take on the role of intergenerational caregivers, allowing women to pursue employment outside the home.

(b) Through stepwise regression, it was found that childcare reduced the probability of employment for married women by 17.27 to 23.67%. The conclusion is consistent with existing typical study (Viitanen, 2005). Rural women’s choices between caring for children and working depend on their evaluation of the expected outcomes and values of these two behaviors. When rural women assess the expected outcomes of caring for children, they may consider factors such as the emotional wellbeing and development of their children, the quality of the parent-child relationship, and the sense of fulfillment derived from being actively involved in their children’s lives.

(c) By constructing a moderation effect model, the presence of kindergarten facilities had a significant positive effect on rural women’s employment, increasing it by 3.97%. The conclusion is consistent with existing typical study (Cascio, 2009). According to the social support theory, kindergarten, as public services, provides women with an opportunity to balance their family and work responsibilities. Through the care provided by kindergartens, women can better manage their time and enhance their chances of employment. However, the presence of primary schools did not show any significant mitigation effect on women’s employment constraints. The conclusion is not consistent with existing typical studies (Hand and Baxter, 2013). While primary education is crucial for children’s development, it may not directly address the childcare responsibilities that hinder women’s labor force participation. The lack of an observable effect may be due to various factors, such as the existing division of childcare responsibilities within households or the need for additional support beyond primary school services.

(d) Conducting a grouped regression, it revealed that childcare had a significant negative impact on employment for women aged 20–30, with a reduction of 26.36%. Women aged 31–40 experienced a decrease of 21.82%. Women aged 41–55 experienced a decrease of 5.2%. The conclusion is consistent with existing study (Gu, 2020). According to family responsibility pressure and social expectations, these findings indicate that the age of women plays a crucial role in the relationship between childcare and employment probabilities. It is crucial to provide adequate childcare services and create a supportive environment that enables women to participate in the workforce without compromising their caregiving responsibilities.

(e) When caring for a single child, women experienced a reduction of 8.8% in their employment. When caring for multiple children, women experienced a reduction of 32.8% in their employment. The conclusion is consistent with existing study (Yu and Xie, 2014). The number of children directly affects rural women’s job opportunities. In rural areas, due to the lack of appropriate childcare facilities and support systems, women have to personally take care of their children. It is crucial for the government and social organizations to provide appropriate support and resources to assist rural women in their work, thereby overcoming the challenges posed by family structure and the number of children.

(f) Childcare led to a decrease of 18.40% in women’s employment from low-income households. For high-income households, there was an increase of 18.75% on women’s employment. The impact was minor in middle-income households. The results revealed varying effects based on income levels. The conclusion is consistent with existing study (Lundberg and Rose, 2002). The results align with theories that highlight the importance of economic factors in shaping women’s employment opportunities. Low-income households may face greater financial constraints, which can hinder women’s employment. Conversely, high-income households may have more resources, leading to increased employment for women. Factors other than childcare, such as skills or occupational choices, may play a role in shaping employment for women in middle-income households.

## 8. Conclusion

Family care is an impediment to rural Chinese women’s employment. This paper provided a new perspective on releasing China’s rural female labor force for increasing market participation rates. As China’s formal care system was introduced and rural public service facilities were enhanced, it played an important and positive role in promoting female labor force participation in the labor market. However, as the age of the female labor force, the number of children, and the level of household income vary, new challenges will be posed to female labor force participation. The main results are as follows:

This paper found that rural women facing childcare pressures did indeed have a reduced likelihood of employment. This likelihood was influenced by factors such as age, the number of children, and family income in rural areas. Among low-income families, women aged 20–30 with multiple children faced the greatest employment constraints. The presence of kindergartens reduced the constraints of childcare and increased the potential for employment. A surprising finding was that caring for elderly individuals with functional abilities did not significantly reduce women’s motivation and likelihood of work. Another unexpected finding is that once there were primary school-aged children in the household, it reduces the likelihood of rural women’s employment. They chose to spend time with their children for the purpose of education. This raised a deeper consideration that rural women are, in fact, the “teachers” of their own children.

To alleviate the constraints of family care and enhance rural women’s labor force participation, efforts can be made in the following aspects:

First, it is necessary to enhance the protection of women’s labor force participation rights and associated benefits, such as setting age limits for recruitment and providing maternity leave during employment. By safeguarding women’s participation in the labor force and their income, we can prevent gender discrimination in the job market and enhance women’s contribution to household earnings. Second, to alleviate the financial burden on families with multiple children, inclusive childcare services should be actively developed. Infant and childcare services should be included as part of the essential national public services. Additionally, kindergartens should be encouraged to accept children aged 2–3 years, if feasible. The operating hours of childcare facilities should be effectively aligned with women’s working hours. Finally, offering employment arrangements with flexible working hours may be the optimal choice for women to balance family and work responsibilities.

There are some gaps in this paper, which are the directions for further research:

First, for the work-family conflict faced by rural women, this paper only explored the negative effects of family care on women’s employment and how to alleviate this constraint from the perspective of formal caregiving resource supply. Second, the effect of flexible employment on mitigating work-family conflict has only been explored at the theoretical level as well as from the perspective of Interview experience. Limited by the lack of objective data, there is still a lack of empirical evidence to compare the choices between self-employment and employment outside the home for women. In fact, we can design better female employment systems to help women achieve the relationship between family care and self-employment. Finally, the data does not investigate the current state of the market that can accommodate rural women’s employment. It fails to further discuss the job options that can match rural women’s employment. Therefore, future research is yet to explore these areas as well.

## Data availability statement

The original contributions presented in the study are included in the article/supplementary material, further inquiries can be directed to the corresponding author.

## Ethics statement

Ethical review and approval was not required for the study on human participants in accordance with the local legislation and institutional requirements. Written informed consent from the patients/participants or patients/participants’ legal guardian/next of kin was not required to participate in this study in accordance with the national legislation and the institutional requirements.

## Author contributions

XM collected the data, organized the database, wrote the first draft of the manuscript, and performed the statistical analyses with contributions from SW and BH. All authors contributed to the conception and design of the study, manuscript revision, read, and approved the submitted version.
